# Supplementary material for: Wikipedia, friend or foe regarding information on diabetic retinopathy? A content analysis in the world’s leading 19 languages
Source: PLoS One. 2021 Oct 28;16(10):e0258246. doi: 10.1371/journal.pone.0258246 (PMC8553146; doi:10.1371/journal.pone.0258246)
Supplement: S1 File — (DOCX) [file pone.0258246.s001.docx]

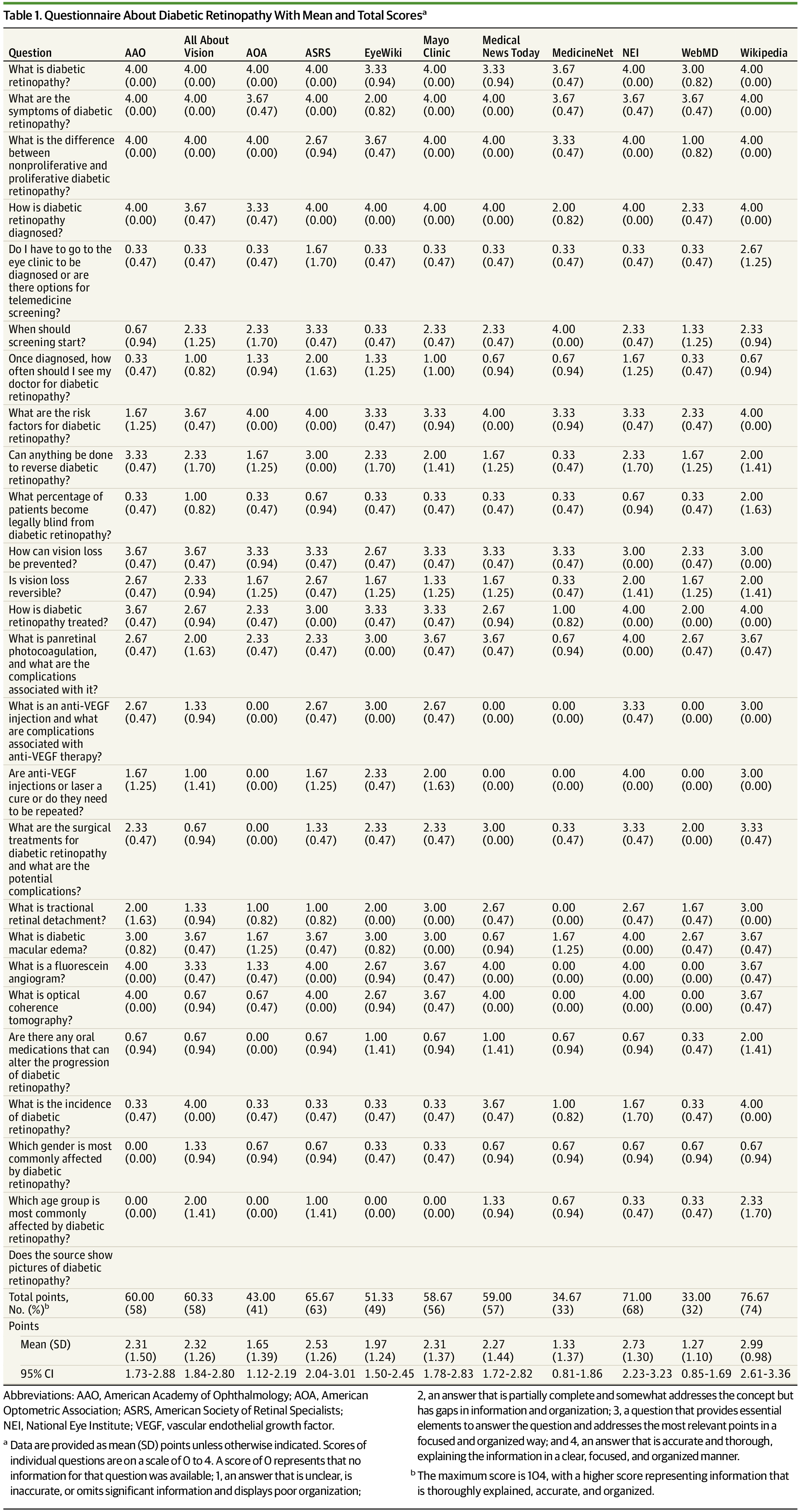
S1 Fig

Questionnaire taken from the following article (open access)

- Kloosterboer A, Yannuzzi NA, Patel NA, Kuriyan AE, Sridhar J. Assessment of the Quality, Content, and Readability of Freely Available Online Information for Patients Regarding Diabetic Retinopathy. JAMA Ophthalmol. 2019;137(11):1240–1245. doi:10.1001/jamaophthalmol.2019.3116

From: **Assessment of the Quality, Content, and Readability of Freely Available Online Information for Patients Regarding Diabetic Retinopathy**

JAMA Ophthalmol. 2019;137(11):1240-1245. doi:10.1001/jamaophthalmol.2019.3116
