# Supplementary material for: Wikipedia, friend or foe regarding information on diabetic retinopathy? A content analysis in the world’s leading 19 languages
Source: PLoS One. 2021 Oct 28;16(10):e0258246. doi: 10.1371/journal.pone.0258246 (PMC8553146; doi:10.1371/journal.pone.0258246)
Supplement: Attachment — Submitted filename: Response to Reviewers.docx [file pone.0258246.s002.docx]

Dear Editor and Reviewers,

Thank you very much for your comments and corrections. We are adding a letter here to explain each point.

We have already checked again that it fulfills the requirements of the journal, as well as the order and format.

Reviewer #1: Thank you for your comments. We have modified the objective of the study with the following: To compare the completeness and quality of information about diabetic retinopathy on Wikipedia in the most spoken languages in the world.

Reviewer #2: Thank you for taking the time to reply to us. With specific regard to the questions we would like to comment that, despite the restriction of Wikipedia in China, we did find a Wikipedia page in that language in the mentioned topic, because we do not exclude Chinese speakers outside the online restrictions of the country.

We explain in the article that our mother tongue is Spanish, but three co-authors are fluent in English, so we were able to understand the literal translations of the content in each language.

In the searching of the term, the results depend on the algorithm of the preferred browser, in this case Google of the previous research to give more consistent results, for example, if we physicians enter a term is more likely to yield scientific pages, but for the average user often the first option in addition to the fact that it is the majority of people are familiar with (not only for medical terms) and is the most widespread, In the articles Kloosterboer A et al 2019 compared the quantity and quality of information on this issue on various websites including (American academy of ophthalmology, Mayo clinic, and others) and concluded that for patient understanding, Wikipedia was the most complete and clearest as well as accurate for the term.

Reviewers #3 and #5: Thank you for your feedback. We already specified in the text and took into account the total number of English speakers including those of us who have English as a second language. We have also included a paragraph with the vulnerabilities and limitations of the study.

Reviewer #4 and #5: Thanks for the great comments; according to Wikipedia´s rules the articles have to have mandatory references for publication as well as a review by the editors of this online encyclopedia. As for the secondary objective, it is to propose that experts on a certain subject should be responsible for modifying the information that is posted on Wikipedia so that it can be updated from a certain time; this is in the discussion section.

Reviewer #6: Thank you very much for the revisions; we recognized the error in the confidence intervals and we corrected them, as well as the order of the tables and paragraphs.
